# Emerging Diagnostic Strategies for Oral Cancer and Oral Potentially Malignant Disorders: A PRISMA-Guided Scoping Review

**DOI:** 10.3390/diagnostics16091364

**Published:** 2026-04-30

**Authors:** Dilara Nur Şengün, Ömer Faruk Kocamaz, Murat Cem Kitap, Merva Soluk Tekkeşin

**Affiliations:** 1 Department of Oral and Maxillofacial Surgery, Faculty of Dentistry, Ankara University, 06560 Ankara, Türkiye; omerf.kocamaz@gmail.com (Ö.F.K.); muratkitap19@gmail.com (M.C.K.); 2 Department of Oral Pathology, Faculty of Dentistry, Ankara University, 06560 Ankara, Türkiye; merva.soluktekkesin@gmail.com

**Keywords:** artificial intelligence, liquid biopsy, oral potentially malignant disorders, oral squamous cell carcinoma

## Abstract

Background/Objectives: Early detection remains the most decisive factor in improving outcomes for oral cancer and oral potentially malignant disorders. However, reliance on conventional biopsy-based pathways presents some practical and biological limitations. This scoping review aimed to map recent advances in non- and minimally invasive diagnostic approaches and to clarify how these innovations are being positioned within clinical workflows. Methods: Following PRISMA-ScR guidance, PubMed/MEDLINE, Scopus, and Web of Science were searched for English-language original studies published between 2020 and 2025. Two independent reviewers screened and charted data on technologies, biomarkers, sampling sources, and clinical applications. Forty-nine studies were included. The literature clustered around four main domains: enhanced cytology (including liquid-based platforms and DNA ploidy analysis), multilayer liquid biopsy strategies (miRNA, cfDNA/ctDNA, methylation panels, and autoantibodies), optical and nanotechnology-based systems (Raman/SERS and sensor platforms), and artificial intelligence-driven decision support tools. Results: Across modalities, a shared emphasis on rapid triage, risk stratification, and follow-up monitoring was evident. Nonetheless, variability in sampling, processing, analytical thresholds, and reporting standards limited cross-study comparability. Conclusions: Recent innovations point toward integrated, panel-based diagnostic models. Broader clinical adoption will require methodological standardization and robust multicenter validation.

## 1. Introduction

Oral cancer, dominated by oral squamous cell carcinoma (OSCC) around 90%, represents a substantial global public health problem. According to the most recent GLOBOCAN 2022 estimates, lip and oral cavity cancer accounted for 389,846 new cases and 188,438 deaths worldwide, ranking 16th and 15th among all cancer sites for incidence and mortality, respectively [1,2,3]. Its high morbidity and mortality rates are largely attributable to delayed diagnosis [4]. Oral potentially malignant disorders (OPMDs), a diverse set of lesions with uncertain malignant transformation potential, account for a significant percentage of OSCC patients [5,6]. Since oral leukoplakia, oral lichen planus, oral lichenoid lesions, and oral submucous fibrosis are known precursors of oral squamous cell carcinoma, early detection of these OPMDs is crucial in determining patient outcomes [7]. Rates of malignant transformation differ significantly among the various OPMD subtypes; proliferative verrucous leukoplakia occurs in approximately 40–50%, while oral lichen planus occurs in less than 2% [8,9]. Despite advances in surgical treatment and adjuvant therapies, the five-year survival rate for OSCC has not changed much in the last few decades; this highlights the importance of early diagnosis and risk assessment [3,10].

The gold standard for traditional diagnostic approaches is still clinical examination, scalpel biopsy, and histopathological examination. However, the decision to perform a biopsy is an invasive method that varies depending on the region and can lead to sampling errors due to tumor heterogeneity [11,12]. This represents a key limitation in the context of tumor heterogeneity, as different regions within the same lesion may exhibit distinct histopathological and molecular characteristics. While multiple biopsies from different sites may improve the assessment of heterogeneity, this approach is not always feasible in routine clinical practice and may increase procedural complexity [13,14]. Considering that repeated biopsies are necessary in the follow-up of OPMDs, these aforementioned difficulties and limitations increase patient burden and healthcare costs [5,15]. Therefore, current technology focuses on developing non-invasive/minimally invasive diagnostic techniques that increase the rate of early diagnosis of these lesions, support individualized clinical decision-making with real-time detection and monitoring [16,17]. In this context, minimally invasive methods, such as brush cytology, saliva- and blood-based liquid biopsy, and optical techniques, allow broader evaluation of molecular and biochemical alterations across lesions, thereby providing additional insight into spatial and dynamic variations of tumor heterogeneity.

In the field of oral oncology, as in other cancer types, a wide variety of new diagnostic methods have been developed. These new methods include liquid biopsy-based molecular analyses using saliva, plasma, or mouthwash samples [18,19,20], advanced cytological techniques such as liquid-based cytology (LBC) and cell blocks obtained from brush biopsies [11,14,21], optical and spectroscopic methods including Raman and surface-enhanced Raman spectroscopy (SERS) [22,23,24], and more recently, artificial intelligence (AI)-supported image and data analysis platforms [25,26,27]. All these technologies aim to overcome the shortcomings of conventional diagnostic methods by detecting molecular, cellular, and phenotypic changes associated with malignant transformation early on and in a minimally invasive manner.

Saliva is a bodily fluid that is easy to collect from the oral cavity and offers a practical advantage for both patients and clinicians, especially when repeated sampling is needed. Due to its constant contact with oral lesions, it also carries diagnostic value and has become an increasingly popular medium in recent research [3,18]. A wide range of salivary biomarkers, including microRNA signatures, cell-free DNA (cfDNA), circulating tumor DNA (ctDNA), DNA methylation patterns, proteomic profiles, and microbiome-derived signals, have revealed promising diagnostic and prognostic potential in oral cancer [4,10,28,29]. Cytology-based approaches have been developed as minimally invasive and cost-effective tools [30]. Recent cytopathology-based research has focused on improving sample quality, enabling access to deeper epithelial layers, and integrating brush cytology with complementary techniques such as image cytometry, DNA ploidy analysis, and immunocytochemical staining [5,6,31]. Furthermore, these methods are supported by machine learning algorithms that can reduce inter-observer variability and enhance diagnostic objectivity [27,32]. Despite these advances, each method has inherent limitations. Cytology-based techniques may be influenced by sampling quality and subjective interpretation, whereas molecular approaches require specialized infrastructure and lack standardized thresholds across studies [33,34,35]. Currently, no single method can be considered definitive; therefore, combined and adjunctive approaches, particularly those integrated with histopathological evaluation, are regarded as the most appropriate strategy for improving diagnostic accuracy in oral cancer.

In addition, optical and nanotechnology-based platforms, including SERS, autofluorescence-assisted cytology, thermal liquid biopsy, and biosensor technologies, have expanded the diagnostic landscape by enabling label-free, rapid, and highly sensitive detection of biochemical alterations associated with oral carcinogenesis [24,36,37,38]. Techniques such as autofluorescence imaging and Raman spectroscopy enable real-time, non-invasive assessment of tissue and cellular composition by identifying molecular signatures related to metabolic and structural changes [39]. SERS and nanotechnology-based platforms further improve sensitivity by amplifying weak molecular signals, allowing the detection of low-abundance biomarkers in biological fluids such as saliva and blood [23,24,38]. Moreover, biosensor technologies offer highly selective and rapid detection of specific biochemical targets, supporting potential point-of-care applications and real-time risk assessment [40]. Importantly, these approaches offer advantages over conventional biopsy by enabling non-invasive and real-time assessment of biochemical changes, potentially allowing earlier detection before morphological alterations become clinically evident. However, despite their promising diagnostic potential, their clinical translation remains limited by challenges related to standardization, reproducibility, and large-scale validation. Therefore, these technologies are best considered as complementary tools that can support, but not replace, histopathological evaluation. The integration of these various methods with AI-based decision support systems represents a significant step toward point-of-care diagnostics and real-time risk assessment in routine dental and medical practice [16,17]. It is important to note that these approaches operate at different levels of the diagnostic process; Raman spectroscopy and its derivatives are analytical data acquisition platforms that produce spectral data reflecting the biomolecular composition of a sample; AI and machine learning algorithms function as data evaluation and pattern recognition tools that can be applied to outputs from any of the above methods.

A review of the literature reveals that despite all technological advancements, significant methodological differences exist in terms of study design, sample types, analytical platforms, and reported findings [3,19]. Most studies focus on proof of concept or early-stage validation; however, there is significant variation among studies regarding clinical applicability, diagnostic performance indicators, and translational capacity [25,26]. Thus, both clinicians and researchers struggle to understand, associate and employ this scattered and complex evidence-based information, where there is no consensus or scheme describing the extent, variability, and clinical relevance of these developing diagnostic technologies.

The aims of this scoping review are: (1) to map genetic and epigenetic biomarkers (somatic mutations in driver genes such as TP53 and DNA methylation panels such as DAPK1 and ZNF471); (2) to characterize transcriptomic markers (salivary miRNAs and extracellular RNAs including miR-196b, miR-133a-3p and miR-375-3p); (3) to evaluate cell-free and circulating nucleic acid approaches (cfDNA, ctDNA) as well as proteomic/autoantibody markers (TK1, NUBP2) in saliva and plasma (4) to identify optical and nanotechnology-based analytical platforms (Raman/SERS spectroscopy, electrochemical biosensors, thermal liquid biopsy); and (5) to evaluate artificial intelligence-based data analysis tools applied to cytological and molecular data. For each domain, clinical advantages and current limitations are discussed in relation to the findings from the included studies. In line with these aims, the research question is structured using the PCC framework: Population: individuals with oral cancer, OPMD, or at risk; Concept: non-invasive and minimally invasive diagnostic methods encompassing molecular, cytological, optical, and AI-based approaches; Context: global clinical and laboratory studies published between 2020 and 2025.

## 2. Materials and Methods

### 2.1. Study Design and Reporting Standards

This study was conducted as a scoping review to systematically map the available evidence, technological diversity, and clinical applications of emerging diagnostic modalities for oral cancer and OPMDs. All methodological stages, from literature search to evidence mapping and synthesis, were planned and conducted in accordance with Preferred Reporting Items for Systematic Reviews and Meta-Analyses extension for Scoping Reviews (PRISMA-ScR). This comprehensive review was not pre-registered in a publicly accessible registry before commencement. However, the eligibility criteria, search strategy, and data planning procedures were defined and documented before data extraction commenced, and the completed PRISMA-ScR checklist is submitted as Supplementary File S1. 

In line with PRISMA-ScR recommendations, the study selection process was structured to identify and categorize relevant evidence rather than to generate pooled estimates. The ‘sample’ in this review therefore refers to the body of included studies, which were systematically identified through database searching and subsequently refined through a two-stage screening process (title/abstract and full-text review). Studies were selected based on their relevance to diagnostic strategies for oral cancer and OPMDs, and were further analyzed according to predefined categories, including diagnostic modality, biomarker type, and sample source (e.g., saliva, cytology, blood-based samples). This approach is consistent with established scoping review methodology, which emphasizes evidence mapping and conceptual categorization over quantitative synthesis [41].

### 2.2. Information Sources and Search Strategy

The research question and scope were structured according to the Population–Concept–Context (PCC) framework:

Population: Individuals diagnosed with oral cancer (including OSCC or OPMDs, as well as individuals at risk).

Concept: Emerging diagnostic modalities, including AI-based approaches, liquid biopsy techniques (circulating free DNA [cfDNA], circulating tumor DNA [ctDNA], microRNA [miRNA]), brush cytology, salivary biomarkers, DNA methylation analysis, and Raman spectroscopy.

Context: Global clinical and laboratory applications published between 2020 and 2025.

In line with this framework, a comprehensive literature search was conducted in PubMed/MEDLINE, Scopus, and Web of Science on 9 February 2026. Studies published between 1 January 2020, and 31 December 2025, were considered eligible.

Given the translational focus of this review, particular attention was given to studies employing minimally invasive and clinically applicable sample types. These primarily included saliva, oral cytology or brush-based samples, oral rinse specimens, and blood-derived samples such as plasma or serum. These sample types were prioritized because they are widely used in contemporary diagnostic research, allow for repeated and non-invasive sampling, and are considered suitable for early detection and disease monitoring. In contrast, studies based solely on conventional tissue biopsy without a clear adjunctive or translational diagnostic component were not the main focus of this review.

The search strategy included combinations of the following keywords: “oral cancer,” “oral potentially malignant disorder,” “artificial intelligence,” “liquid biopsy,” “salivary biomarkers,” and “brush cytology.” The detailed search terms and strategies for each database are presented in Table 1.

### 2.3. Study Selection

Eligibility criteria were developed in accordance with the PCC (Population–Concept–Context) framework defined in Section 2.2. Studies were considered eligible if they directly addressed the defined population (individuals with oral cancer, OSCC, or OPMDs, or those at risk), investigated at least one of the designated concepts (non-invasive or minimally invasive diagnostic modality), and were conducted within the specified context (clinical or laboratory settings). Original full-text research articles published after 1 January 2020, were included in the review. Review articles, case reports, editorials, letters to the editor, and general head and neck studies not directly targeting the oral cavity were excluded. Only studies published in English were considered eligible.

**Figure 1:**
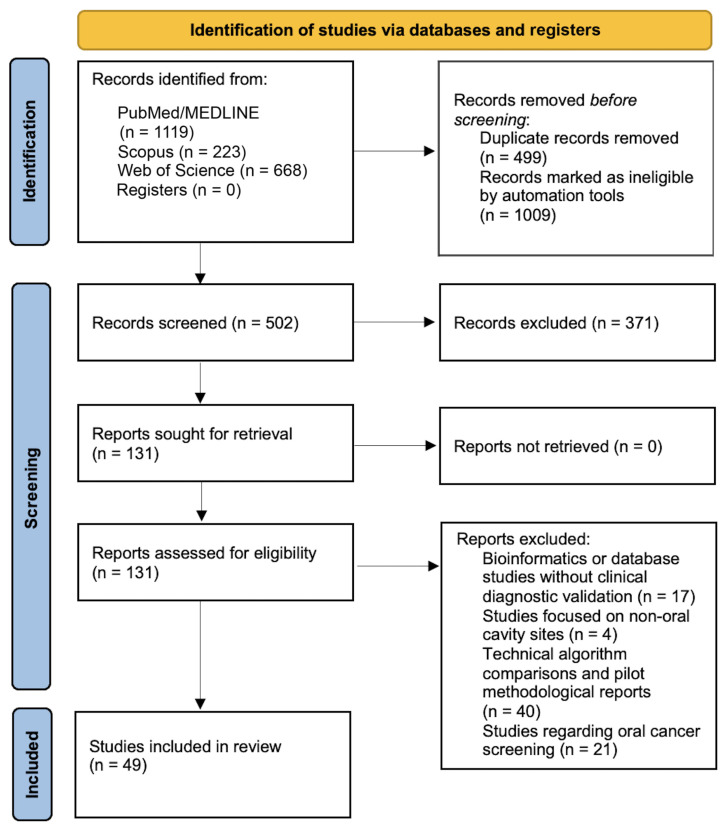
PRISMA flow diagram illustrating the study selection process, including identification, screening, and inclusion stages for studies retrieved from databases and registers.

Title and abstract screening, followed by full-text assessment, were independently performed by two reviewers according to predefined eligibility criteria. Discrepancies were resolved through discussion. As a result of the independent screening process conducted by two reviewers (DS and MST), a total of 49 studies that fully met the inclusion criteria were included in the final analysis. The overall study selection process is illustrated in Figure 1.

Data were charted using a standardized data extraction form developed for this review. The extracted variables included author, publication year, type of technology (e.g., AI, molecular-based, optical modalities), specific biomarker used, sample type (saliva, plasma, brush biopsy), and key clinical outcomes. The data charting process focused on mapping the technological landscape and clinical applications of emerging diagnostic modalities.

## 3. Results

The 49 studies reviewed included a comprehensive examination of innovative modalities in the diagnosis of oral cancer and potentially malignant oral diseases. Table 2 summarizes the details of these studies.

### 3.1. Minimally Invasive Cytological Approaches and Advanced Cellular Analyses

Cytological techniques focus especially on improving sampling quality and strengthening morphological diagnosis with adjuvant analysis. LBC has been shown to reduce cell loss in brush samples, while hard brushes such as Orcellex have been shown to reach deeper epithelial layers and provide higher cellularity than nylon brushes [11,21]. The sensitivity of conventional brush biopsy screening for the detection of cancer cells using brush biopsy in routine clinical practice achieved 100%, and the specificity for the detection of non-neoplastic cells was 86.5%. The positive predictive value was 43.1% and the negative predictive value was 100% [12]. In addition, histopathological comparative studies have shown that different non-invasive cytology techniques can be used safely [54].

DNA image cytometry (DNA-ICM) and DNA ploidy analyses applied on cytological samples to improve diagnostic accuracy provide strong objective data in predicting the risk of malignant transformation of OPMDs [6,31]. At the cellular level, nuclear F-actin phenotyping stands out as a new biomarker in distinguishing dysplastic and malignant cells [63]. Furthermore, immunocytochemical markers applied in cell blocks obtained from brush cytology are helpful in determining the biological behavior of lesions [5,49]. ‘Cytomics-on-a-chip’ platforms based on microfluidic systems allow rapid risk stratification at the bedside [17].

### 3.2. Salivary Biomarkers and Multilayer Liquid Biopsy

Liquid biopsy studies proceed from three main branches: miRNA panels, somatic mutations and epigenetics. Specific miRNA biomarkers isolated from salivary exosomes or serum (e.g., miR-196b, miR-375, miR-133a-3p) have shown diagnostic potential for oral cancer and OPMDs [4,10], while specific miRNA networks have also shown potential for predicting disease progression and survival [50]. The identification of novel RNA markers, such as CLEC2B, in saliva suggests that saliva may be superior to plasma in non-invasive diagnosis [18]. It is emphasized that it is possible to identify somatic mutations in driver genes in saliva collected during the diagnosis of OSCC [47].

Molecular follow-up found that extracellular DNA (cfDNA) and mitochondrial DNA (cf-mtDNA) levels in plasma and saliva were directly related to tumor burden [20,59]. In particular, the follow-up of TP53 somatic mutations with ultra-sensitive NGS methods serves as a critical ‘molecular monitor’ in evaluating the adequacy of surgical resection margins and early detection of recurrences [43,45,47,62]. At the epigenetic level, multigene DNA methylation panels (DAPK1, ZNF471, etc.) in saliva exhibit a specificity of over 90% in the diagnosis of early-stage tongue cancers [29,57]. Furthermore, ctDNA has been shown to be a valuable non-invasive prognostic biomarker for monitoring tumor burden, assessing treatment response, and predicting survival in OSCC patients [61].

Autoantibodies such as thymidine kinase 1 (TK1), nucleotide-binding protein 2 (NUBP2) and protein pyrroline-5-carboxylate reductase 1 (PYCR1), which were examined between OSCC and control groups, have been shown to be used as potential biomarkers in determining disease-free survival [28].

### 3.3. Optical Spectroscopy and Nanotechnological Sensors

Raman and SERS technologies enable ‘label-free’ diagnosis by removing the chemical fingerprint of biological samples. Raman and SERS analyses in salivary exosomes and serum provide high diagnostic accuracy in OSCC and oropharyngeal cancers [23,64]. As a nanotechnological innovation, early-stage diagnosis has become possible with the detection of volatile components in exhaled breath using Ag NWs@ZIF core–shell nanochains [38]. Furthermore, spectroscopic fusion of plasma and saliva offers higher success rates compared to single assays [24]. Thermal liquid biopsy based on physical characteristics-based differential scanning calorimetry (DSC) and biosensors developed for TGF-β1 detection are other modalities that accelerate the diagnostic process [36,37]. 

### 3.4. Artificial Intelligence and Decision Support Systems

AI plays a key role in the processing of digital pathology and radiomic data. Deep learning models using the autofluorescence feature of Papanicolaou dye can automatically detect cancer cells in cytopathological scans [32]. McRae et al. [16] developed a point-of-care oral cytology tool that analyzes cell phenotypes from brush cytology samples using a cytology-on-a-chip method in a large cohort of 999 patients with AI-based machine learning algorithms, achieving AUC values of 0.88 for binary low-risk versus high-risk and 0.92 for moderate versus severe dysplasia, with cell phenotype classification accuracy of 99.3%. In addition, machine learning algorithms reduce subjectivity in diagnosis with automatic analysis of nuclear parameters and quantification of nucleolar organizer regions (NORs) [26,27].

## 4. Discussion

Oral cavity cancers still carry a significant incidence and mortality burden worldwide and the most important factor determining the prognosis is the detection of the disease at an early stage [2]. However, early diagnosis in the spectrum of oral cancer and OPMDs can be difficult due to the clinical similarity of the lesions, the variability in dysplasia grading, and the practical limitations of invasive biopsy [7,65]. In recent years, the number of studies aimed at overcoming this difficulty has increased significantly. In particular, the strengthening of minimally invasive cytology with advanced cellular analysis, the widespread use of saliva/plasma-based liquid biopsy approaches, the use of optical techniques such as Raman/SERS, and the introduction of AI-supported decision systems suggest that diagnostic approaches are in a rapid transformation. This scoping review maps the studies published in the 2020–2025 period in line with the PRISMA-ScR principles. The objective of this scoping review is to reveal the clinical and laboratory application areas of these technologies and the heterogeneity in the literature in a holistic manner.

Minimally invasive cytology provides the clinician with both a rapid and low-morbidity assessment in the suspicion of oral cancer and OPMDs. A notable point in recent years is that the method has moved away from a “simple morphology” approach and has been strengthened with steps that improve sampling quality and make the results more objective. LBC offers a practical advantage in this respect; furthermore, it has been shown that brush design can significantly affect sample adequacy, with higher cellularity being achieved with more rigid brushes such as Orcellex [11]. It is also important for daily practice that LBCs can be reported in a manner consistent with standardized cytology classifications [21]. Nevertheless, performance data from routine clinical settings suggest that the principal strength of the method may lie in its reassuring value when results are negative. Neumann et al. reported that while sensitivity and negative predictive value can be relatively high, the positive predictive value may remain limited [12]. Comparative studies with histopathology support the idea that different non-invasive cytology techniques can find a place in clinical practice with appropriate case selection [54]. In summary, cytology is currently positioned not as an alternative to biopsy in most scenarios, but rather as a “triage” step that more rationally directs suspicious lesions towards biopsy. This framework is consistent with both the Cochrane review and more recent reviews, which emphasize that while adjunctive diagnostic tests are promising, they have not yet replaced histopathology due to heterogeneity and lack of external validation [30,66]. Recent reviews focusing on LBC similarly indicate that standardization has strengthened, but training and multi-center validation are still needed [67]. Moreover, the fact that cytology alone is insufficient to predict biological behavior, especially in borderline lesions, has underscored the need to include additional biological layers in the evaluation, and it has been observed that quantitative and functional data obtained from cytological samples can add depth to the diagnostic process. In this regard, DNA image cytometry (DNA-ICM) and DNA ploidy analyses contribute to predicting the risk of malignant transformation in oral potential malignant disorders by measurably revealing genomic instability at the cellular level [6,31]. Complementing these quantitative approaches, nuclear F-actin phenotyping has been identified as an additional biomarker that goes beyond morphological assessment in differentiating dysplastic and malignant cells. Similarly, it has been reported that immunocytochemical markers applied to cell blocks obtained from brush cytology can help to interpret the biological behavior of lesions in more detail [5,49,63].

The growing body of evidence in the field of liquid biopsy over recent years indicates a clear shift away from the search for a single diagnostic biomarker toward integrative approaches that simultaneously interrogate multiple biological layers in the detection of oral cancer and OPMDs. miRNA profiles are the most visible part of this line; miRNA signatures derived from saliva exosomes or serum are reporting promising accuracy in distinguishing OSCC cases from healthy individuals, and retesting of candidates, particularly miR-196b, in different cohorts suggests that the findings are becoming increasingly “settled” [10]. Similarly, studies comparing saliva and blood-derived extracellular RNAs have shown that some transcripts (e.g., CLEC2B) show more pronounced separation in saliva, supporting the idea that saliva may be a more practical and even more sensitive sample source than plasma in some clinical scenarios [18]. With regard to somatic mutations, new data suggests that driver gene mutations can be detected in saliva samples taken at the time of diagnosis with ultra-sensitive NGS [47]. These data indicate that liquid biopsy can produce value not only in diagnosis but also in clinical translation, such as postoperative margin adequacy and early recurrence monitoring. This approach also seems consistent with new studies on the traceability of TP53-mutated tumor DNA in plasma and saliva [62]. Findings reporting that cfDNA and cf-mtDNA levels can be correlated with tumor burden [20] suggest that simpler, measurable monitoring metrics can be developed. On the epigenetic side, multigene methylation panels (DAPK1, ZNF471, etc.) and novel methylation biomarkers in salivary DNA stand out with high specificity target in early-stage tongue cancer [57,68]. When reviews summarizing the relationship of ctDNA/cfDNA with prognosis and treatment response [61] and alternative approaches such as autoantibody profiling [28] are considered together, it is understood that the field is gradually shifting towards a panel/integration logic. In this context, the demonstration that some autoantibodies—e.g., TK1, NUBP2 and PYCR1—evaluated between OSCC patients and control groups may be associated with disease-free survival,= suggests that liquid biopsy has potential not only for diagnosis but also for prognosis and monitoring [28]. Indeed, recent systematic reviews evaluating salivary miRNAs highlight the considerable potential; however, heterogeneity persists due to differences in sampling and processing steps, analysis thresholds, and study designs, and standardization and external validation are still needed for clinical dissemination [46,69]. ctDNA/cfDNA are genetic/mutation-based biomarkers that exhibit high specificity for tumor-derived signals. However, ctDNA levels tend to be low in early-stage oral lesions, which can cause limiting sensitivity [42]. Epigenetic markers show high specificity in distinguishing high-risk OSCC groups from normal tissue and are less susceptible to contamination than mutation-based approaches, but quantitative thresholds for clinical decision-making have not yet been standardized [35,42]. Transcriptomic markers readily detectable in saliva, miR-196b and miR-133a-3p/miR-375-3p, have shown consistent results in independent cohorts [4,10]. However, pre-analytical variability (sampling protocols, storage conditions, etc.) limits inter-study comparability. Proteomic/autoantibody markers, which have the potential for non-invasive monitoring of treatment response and disease-free survival, are currently limited due to the lack of validated threshold values and small cohort sizes [28].

Sampling method and processing media also critically impact diagnostic performance. Among brush types, rigid brushes (e.g., Orcellex) have been associated with higher specificity compared to standard nylon brushes, possibly due to more effective sampling of deeper epithelial layers [11]. Regarding processing media, liquid-based cytology platforms have demonstrated advantages over conventional exfoliative cytology by reducing masking elements, improving cellular protection, and enabling auxiliary molecular analyses on residual material. Additionally, mouthwash/mouth rinse samples appear more suitable for DNA methylation analysis and ctDNA retrieval in liquid biopsy applications [44], while saliva shows comparative advantages for miRNA and extracellular RNA profiling [10]. Plasma-based approaches offer lower background contamination for cfDNA/ctDNA but may reduce sensitivity in early-stage or localized lesions where tumor release into the systemic circulation is minimal.

The sampling and laboratory infrastructure requirements of biomarker-based approaches have increased interest in faster and ‘label-free’ solutions in the clinic; optical spectroscopy and sensor-based platforms have emerged with the aim of responding to this need [22,24].

Optical spectroscopy and nanotechnology sensors have recently attracted attention, particularly for their promise of “label-free” and rapid analysis in oral cancer diagnosis. Studies demonstrating that SERS spectra can provide discriminatory power through multivariate analysis via saliva exosomes reveal the applicability of this approach in differentiating oral cavity and oropharyngeal SCC cases from healthy individuals [23]. Similarly, methodological improvements focusing on addressing pre-sample analytical issues (e.g., hemolysis) in serum Raman/SERS data aim to bring classification performance closer to clinical conditions [64]. As a more exploratory innovation, the capture of volatile biomarkers in exhaled breath using Ag NWs@ZIF core–shell nanochains, followed by AI-based classification, extends the concept of non-invasive screening into an entirely new domain [38]. In addition, a “spectroscopic fusion” approach—integrating spectral data from both plasma and saliva rather than relying on a single biofluid—has been reported to yield greater clinical value compared with individual analyses alone [24]. Beyond spectroscopy, sensor-based innovations such as DSC-based “thermal liquid biopsy” (used for saliva and plasma) and molecularly imprinted polymer (MIP)-based electrochemical biosensors developed for TGF-β1 have the potential to accelerate the diagnostic process [36,37]. When this technological spectrum is considered alongside point-of-care “oral cytology devices” evaluated in large cohorts and machine learning–based risk stratification models [16], a common objective becomes apparent: the effort to address the need for rapid and practical triage tools in routine clinical practice. Similarly, it is reported that “cytomics-on-a-chip” platforms based on microfluidic systems can enable rapid and holistic risk classification in the clinic by evaluating cytological and cellular parameters together on the same sample [17]. Still, recent reviews highlight that Raman/SERS is a strong candidate for being an adjunctive diagnostic tool, but their translation into routine clinical practice will remain limited until standardization, external validation, and real-world implementation steps are completed [70].

It is obvious that label-free and rapid, multi-fluid fusion approaches (plasma + saliva) with optical/spectroscopic platforms (Raman/SERS) that do not require complex sample preparation improve accuracy. However, barriers to clinical implementation include high equipment costs, operator expertise requirements, and the lack of standardized spectral libraries. In addition, a shared challenge lies in translating the growing volume of data into actionable clinical decisions. For both spectroscopic outputs and cytological or im-age-based datasets, AI has emerged as a critical complementary tool, transforming raw data into clinically meaningful classifications.

The role of AI in this field should be conceptually separated from the analytical methods it processes. It does not generate new biological signals; instead, AI-based algorithms trained to recognize distinctive structures within existing data enable the classification of complex spectral patterns that are undetectable by human analysts in the context of Raman/SERS spectroscopy. In cytopathology, deep learning models identify morphological and textural features in stained cell images. In liquid biopsy, artificial intelligence integrates multiple biomarker panels to improve overall diagnostic accuracy beyond a single biomarker threshold. A hierarchical relationship exists here. The analytical platform receives the data, performs AI-based interpretation, and represents the emerging integrated paradigm in oral cancer diagnosis.

AI–based decision-support approaches have gained prominence particularly in cytology and digital pathology, with the aim of rendering evaluations more standardized and reproducible. Deep learning models that also leverage the autofluorescent properties of Papanicolaou staining have demonstrated the ability to automatically detect malignant cells in cytopathological screenings [32]. Similarly, automated analysis of nuclear parameters in exfoliative cytology has the potential to strengthen the “triage” approach by reducing observer-dependent variations [27]. Studies demonstrating that AgNOR quantification can be performed with AI also indicate that this proliferation-associated marker can be used in a more objective and standardized manner [26]. However, for these models to become established practices, external validation with data from different centers, standardization of imaging/labeling processes, and more transparent reporting steps that will allow clinicians to interpret the decision with confidence still seem crucial.

Beyond the biological properties of the biomarker, the choice of analytical platform significantly determines diagnostic performance. Droplet digital PCR (ddPCR) for methylation detection in oral rinses shows potential for detection and monitoring of oral cancer [44,47]. Next-generation sequencing with unique molecular identifiers further reduces technical noise and enables the detection of tumor-specific somatic mutations with high confidence [47]. For spectroscopic methods, multivariate data analysis algorithms applied to Raman spectral data directly determine classification accuracy; methodological preferences regarding preprocessing and spectral range selection critically affect reported performance metrics [24]. Training dataset size, image resolution, staining consistency, and model architecture, which affect both sensitivity and specificity, are critical for AI/deep learning models applied to cytological images. Models trained on single-center data, in particular, routinely experience performance degradation after external validation; this clearly highlights the need for training cohorts with multiple center studies.

The studies reviewed reported promising diagnostic accuracy figures, many showing sensitivity or accuracy above 85–90%. For example, Raman microspectroscopy by Behl et al. [22] showed sensitivity above 90% in dysplastic lesions. Koster et al. [24] achieved 91.7% accuracy with combined plasma and saliva Raman spectroscopy. Castillo et al. [48] achieved 88% sensitivity for HPV detection with liquid-based brush cytology. Gaida et al. [11] showed that the rigid (Orcellex) brush achieved higher sensitivity (95.6%) compared to the nylon brush; however, it also produced a higher proportion of doubtful diagnoses (11.1% vs. 5.4%), which may negatively affect specificity. McRae et al. [63] demonstrated that lasso-logistic regression models incorporating nuclear F-actin cytological signatures could differentiate between early (benign vs. more severe lesions) and late-stage (moderate/severe dysplasia and carcinoma vs. less severe lesions) oral diseases with AUC values of 0.82 and 0.93, respectively, and concluded that cytological features substantially improved upon lesion appearance and risk factors in predicting OSCC. Neumann et al. [12] reported that although sensitivity (100%) and negative predictive value (100%) were high, positive predictive value (43.1%) remained limited. Direct comparison is not possible due to these heterogeneous reference standards, varying patient selection criteria, and inconsistent reporting of confidence intervals and ROC/AUC values.

While it is easier to discuss and evaluate such approaches in the context of oral SCC, attempting to characterize OPMDs, which involve fundamentally different etiopathogenesis and diverse molecular landscapes, is far more complex. Oral leukoplakia, the most common OPMD, is an epithelial dysplastic disorder, and the relevant biomarkers reflect epithelial cell cycle dysregulation, genomic instability (e.g., DNA ploidy, TP53 mutations), and epigenetic silencing of tumor suppressor genes. In contrast, oral lichen planus is an immune-mediated condition primarily driven by cytotoxic CD8+ T-lymphocyte activation, and its relevant biomarkers are largely lymphocyte-derived, making the application of standard tumor-derived molecular biomarkers to oral lichen planus less feasible. Oral submucosal fibrosis involves progressive fibrosis of the submucosal connective tissue, and relevant markers include TGF-β1, collagen cross-linking enzymes, and fibroblast activation markers. Subtype-specific biomarker panels for OPMDs are another urgent need.

## 5. Conclusions

In conclusion, this scoping review demonstrates that between 2020–2025, there has been a rapid diversification of approaches in the diagnosis of oral cancer and OPMDs, focusing around minimally invasive cytology, liquid biopsy, optical/nanotechnological platforms, and AI-based approaches. However, current studies reveal a significant heterogeneity in terms of sampling-processing steps, analysis thresholds and reporting methods, which limits the clinical generalizability of the findings. Accordingly, the next critical step involves expanding multicenter external validation studies conducted under standardized protocols, while also clarifying in which specific clinical context screening, triage, or surveillance-integrated, panel-based approaches across different modalities can deliver the greatest clinical value.

## Figures and Tables

**Table 1:** Search strategies used in each database.

Data Base	Search Strategies
PubMed/MEDLINE	(oral cancer diagnosis OR oral potentially malignant disorder OR oral potentially malignant disease) AND (artificial intelligence OR digital pathology OR brush cytology OR (salivary biomarker AND dna methylation OR liquid biopsy))
Scopus	ALL ((oral cancer diagnosis OR oral potentially malignant disorder OR oral potentially malignant disease) AND (artificial intelligence OR digital pathology OR brush cytology OR (salivary biomarker AND dna methylation OR liquid biopsy)))
Web of Science	((ALL = (oral cancer diagnosis OR oral potentially malignant disorders OR oral potentially malignant diseases)) AND (ALL = (artificial intelligence OR digital pathology OR brush cytology OR (salivary biomarkers AND dna methylation OR liquid biopsy))))

**Table 2:** Overview of emerging minimally invasive and technology-based diagnostic approaches for oral cancer and OPMDs (2020–2025).

Authors	Publication Year	Type of Technology	Specific Biomarker Used	Sample Type	Key Clinical Outcomes
Behl, I; Calado, G; Malkin, A; Flint, S; Galvin, S; Healy, CM; Pimentel, ML; Byrne, HJ; Lyng, FM [22]	2020	Raman micro-spectroscopy		Brush biopsy	Sensitivity exceeding 90% in early-stage lesions.
Kondo, Y; Oya, K; Sakai, M; Fujiwara, C; Tojo, F; Usami, Y; Fukuda, Y; Kogo, M; Kishino, M [21]	2020	liquid-based cytology		liquid-based cytology (conventional exfoliative cytology (CEC) with LBC and LBC alone)	High diagnostic concordance of LBC in the oral cavity.
Gissi DB, Gabusi A, Tarsitano A, Asioli S, Rossi R, Marchetti C, Montebugnoli L, Foschini MP, Morandi L [42]	2020	molecular-based	13-gene panel	non-invasive oral brushing	DNA methylation is elevated only in high-risk OSCC groups and may serve as a reliable oral cancer risk indicator.
Crimi, S; Falzone, L; Gattuso, G; Grillo, CM; Candido, S; Bianchi, A; Libra, M [4]	2020	molecular-based and liquid biopsy	hsa-miR-133a-3p hsa-miR-375-3p	Droplet Digital PCR Analysis of Liquid Biopsy	miR-133a-3p and miR-375-3p are strong diagnostic markers involved in oral cancer pathogenesis and aggressiveness.
McRae, MP; Modak, SS; Simmons, GW; Trochesset, DA; Kerr, AR; Thornhill, MH; Redding, SW; Vigneswaran, N; Kang, SK; Christodoulides, NJ; Murdoch, C; Dietl, SJ; Markham, R; McDevitt, JT [16]	2020	machine learning		cytology-on-a-chip approach	A POC-compatible cytology platform demonstrates screening capability, and F-actin phenotype determines dysplasia grade.
Husain, A; Singhal, A; Agarwal, A; Hadi, R; Husain, N [43]	2020	molecular-based	specific detection of circulating tumor DNA (ctDNA)	plasma	ctDNA levels were lower in chronic smokers, and postoperative decline indicates adequacy of surgical resection.
Gaida, K; Deuerling, L; Neumann, H; Remmerbach, TW [11]	2021	liquid-based cytology		liquid-based brush	Both systems showed similar sensitivity, but the rigid Orcellex brush demonstrated superior specificity over nylon brushes.
Fung, SYH; Chan, KCA; Wong, EWY; Ng, CWK; Cho, R; Yeung, ZWC; Lam, JWK; Chan, JYK [44]	2021	molecular-based	Tumor suppressor gene methylation	oral rinse	Tumor suppressor gene methylation monitoring via ddPCR enables sensitive diagnosis and post-treatment surveillance in HNSCC.
Kujan, O; Idrees, M; Anand, N; Soh, B; Wong, EL; Farah, CS [5]	2021	molecular-based	immunocytochemistry of DNA mismatch repair proteins	oral brush cytology	Cell block ICC improves diagnostic accuracy and is effective in diagnosing oral leukoplakia.
Cui Y, Kim HS, Cho ES, Han D, Park JA, Park JY, Nam W, Kim HJ, Cha IH, Cha YH [45]	2021	molecular-based	Tumor-specific mutations	saliva and plasma	Postoperative ctDNA SNVs predicted recurrence, enabling detection nearly four months before clinical diagnosis.
McRae, MP; Kerr, AR; Janal, MN; Thornhill, MH; Redding, SW; Vigneswaran, N; Kang, SK; Niederman, R; Christodoulides, NJ; Trochesset, DA; Murdoch, C; Dapkins; Bouquot, J; Modak, SS; Simmons, GW; McDevitt, JT [46]	2021	molecular-based	Nuclear F-actin	Oral brush biopsy	Lasso logistic regression achieved high accuracy, with cytological signatures outperforming clinical features in early and late diseases
Cheng, AJ; You, GR; Lee, CJ; Lu, YC; Tang, SJ; Huang, YF; Huang, YC; Lee, LY; Fan, KH; Chen, YC; Huang, SF; Chang, JTC [10]	2021	molecular-based	miR-196b	saliva	Salivary miR-196b shows strong potential as a malignant transformation signal in OPMDs and high-risk HNSCC screening.
Shanmugam, A; Hariharan, AK; Hasina, R; Nair, JR; Katragadda, S; Irusappan, S; Ravichandran, A; Veeramachaneni, V; Bettadapura, R; Bhati, M; Ramaswamy, V; Rao, VUS; Bagadia, RK; Manjunath, A; Manjunath, NML; Solomon, MC; Maji, S; Bahadur, U; Bettegowda, C; Papadopoulos, N; Lingen, MW; Hariharan, R; Gupta, V; Agrawal, N; Izumchenko, E [47]	2021	molecular-based	Tumor-Specific Mutations	saliva	UMI-based dual-capture sequencing enables ultra-sensitive detection of tumor-specific somatic mutations in saliva.
Castillo, P; de la Oliva, J; Alos, S; Perez, F; Vega, N; Vilaseca, I; Marti, C; Ferrer, A; Alos, L [48]	2022	liquid-based brush cytology		liquid-based brush cytology	Brush cytology achieved 88% sensitivity for malignancy diagnosis before and after treatment.
Koster, HJ; Guillen-Perez, A; Gomez-Diaz, JS; Navas-Moreno, M; Birkeland, AC; Carney, RP [24]	2022	Raman spectroscopic		blood and saliva	Combined plasma and saliva analysis achieved 91.7% accuracy, confirming Raman spectroscopy sensitivity to cancer-related metabolites.
Idrees, M., Shearston, K., Farah, C.S., Kujan, O [49]	2022	machine learning		oral brush biopsy	AI-assisted cell-block immunostaining analysis enables reliable molecular differentiation of OLP and lichenoid lesions.
Adeoye, J; Wan, CCJ; Zheng, LW; Thomson, P; Choi, SW; Su, YX [29]	2022	molecular-based-Machine Learning	DNA Methylation Analysis	salivary	Machine learning–based DNA methylation mapping achieved perfect sensitivity and specificity in distinguishing oral cancer from OPMDs.
Idrees, M; Farah, CS; Sloan, P; Kujan, O [15]	2022	modified 2014 Bethesda cytology system		Oral brush biopsy	OLBC achieved 91.69% accuracy and is 26–42% more cost-effective than biopsy for malignancy risk stratification.
Neumann, FW; Neumann, H; Spieth, S; Remmerbach, TW [12]	2022	oral brush biopsy		oral brush biopsy	Oral brush biopsy demonstrated 100% sensitivity for detecting cancer cells in routine dental practice.
Piao, Y; Jung, SN; Lim, MA; Oh, C; Jin, YL; Kim, HJ; Nguyen, QK; Chang, JW; Won, HR; Koo, BS [19]	2023	molecular-based	circulating microRNA	plasma	Combined miRNAs (AUC = 0.899) improved accuracy; miR-92a-3p/92b-3p serve as dynamic monitors in OSCC follow-up.
Faur, CI; Dinu, C; Toma, V; Jurj, A; Marginean, R; Onaciu, A; Roman, RC; Culic, C; Chirila, M; Rotar, H; Falamas, A; Stiufiuc, GF; Hedesiu, M; Almasan, O; Stiufiuc, RI [23]	2023	Raman micro-spectroscopy		salivary	Multivariate SERS analysis of salivary exosomes enables rapid and improved OSCC detection.
Patel, A; Patel, P; Mandlik, D; Patel, K; Malaviya, P; Johar, K; Swamy, KBS; Patel, S; Tanavde, V [50]	2023	molecular-based	3-miRNA	tissue and salivary	The 3-miRNA signature (AUC 0.99) regulates OSCC progression and predicts survival and recurrence.
Banavar, G; Ogundijo, O; Julian, C; Toma, R; Camacho, F; Torres, PJ; Hu, L; Chandra, T; Piscitello, A; Kenny, L; Vasani, S; Batstone, M; Dimitrova, N; Vuyisich, M; Amar, S; Punyadeera, C [51]	2023	molecular-based	RNA	salivary	Host–microbiome signature–based screening shifted 27% of late-stage oral cancers to early-stage detection.
Siciliano, G; Chiriaco, MS; Ferrara, F; Turco, A; Velardi, L; Signore, MA; Esposito, M; Gigli, G; Primiceri, E [37]	2023	molecular-based	TGF-β1	liquid biopsy	A low-cost sensor for TGF-β1 used optimized electropolymerization but 3 scans were unstable, while 10 scans hindered template removal.
Ribeiro, MGM; Dolabella, SS; Trento, CL; Barros, JD; Freitas, VS; Daltoe, FP; Grando, LJ; Machado, MJ; Onofre, FBD; Onofre, ASC [31]	2023	molecular-based	DNA-ICM	oral brush biopsy	Combined brush cytology and DNA-ICM achieved 81.4% sensitivity and 90.9% specificity.
Xie, X; Yu, WR; Chen, ZX; Wang, L; Yang, JJ; Liu, SH; Li, LZ; Li, YX; Huang, YZ [38]	2023	artificial intelligence-Raman spectroscopy	Ag NWs@ZIF core–shell nanochains	exhaled breath	Ag NWs@ZIF nanowire-based SERS substrate enables enhanced Raman signal and supports early cancer detection.
Kokubun, K; Nakajima, K; Yamamoto, K; Akashi, Y; Matsuzaka, K [14]	2023	oral liquid-based cytological		oral brush biopsy	Despite cytology limitations, histological concordance showed a 92% negative predictive value in a large cohort.
Lin, LH; Chang, KW; Cheng, HW; Liu, CJ [52]	2023	molecular-based	Somatic Mutations in Plasma Cell-Free DNA	liquid biopsy	Cytology and DNA-ICM showed 81.4% sensitivity and 90.9% specificity, enabling somatic mutation burden monitoring in metastatic OSCC.
Chaudhuri, D., Ghosh, A., Raha, S., Chakraborty, A., Chatterjee, K., Barui, A [53]	2023	Raman spectroscopy/machine learning		Raman spectroscopy	Raman spectroscopy applications are rapidly increasing, achieving 88% diagnostic accuracy with MLA ensembles.
Sayal, L; Hamadah, O; Almasri, A; Idrees, M; Thomson, P; Kujan, O [20]	2023	molecular-based	cell-free DNA and cell-free mitochondrial DNA	saliva	Salivary cfDNA and cf-mtDNA levels were significantly elevated, supporting mitochondrial DNA as an early diagnostic marker.
Abdulla, R; Puthenpurackal, JD; Pinto, SM; Rekha, PD; Subbannayya, Y [28]	2023	molecular-based	These included autoantibodies against Thymidine kinase 1 (TK1), nucleotide-binding protein 2 (NUBP2), and protein pyrroline-5-carboxylate reductase 1 (PYCR1)	serum	TAAbs are stable early biomarkers. NUBP2 autoantibody shows potential for evaluating OSCC survival.
Kumarguru, BN; Narayana, ML; Urvashi, G; Ramaswamy, AS [54]	2023	brush cytology/scrape cytology		brush cytology/scrape cytology	Brush cytology shows better histopathological correlation than scrape cytology, supporting a more reliable noninvasive diagnostic approach.
Mcrae, MP; Rajsri, KS; Kerr, AR; Vigneswaran, N; Redding, SW; Janal, M; Kang, SK; Palomo, L; Christodoulides, NJ; Singh, M; Johnston, J; Mcdevitt, JT [17]	2024	scalpel biopsy/brush cytology		scalpel biopsy/brush cytology	The brush cytomics model improved diagnostic performance, achieving an AUC of 0.76 for OLC risk stratification.
Balakittnen, J; Weeramange, CE; Wallace, DF; Duijf, PHG; Cristino, AS; Hartel, G; Barrero, RA; Taheri, T; Kenny, L; Vasani, S; Batstone, M; Breik, O; Punyadeera, C [3]	2024	molecular-based	mi-RNA	saliva	The 8-miRNA signature predicted cancer risk in OPMDs with AUC 0.954, 86% sensitivity, and 90% specificity.
Mhaske, S; Ramalingam, K; Nair, P; Patel, S; Menon, PA; Malik, N; Mhaske, S [27]	2024	Machine Learning/nuclear parameters		Oral Exfoliative Cytology	Machine learning–based automated nuclear analysis improves accuracy and speed in oral cancer cytology diagnosis.
Koriakina N, Sladoje N, Bašić V, Lindblad J [25]	2024	machine learning		oral brush biopsy	It is possible not only to separate healthy patients from patients with malignancy, but also to detect cells related to malignancy, by utilizing only patient-level annotations on a fairly small number of patients.
Schneider, G; Kaliappan, A; Joos, N; Dooley, LM; Shumway, BS; Chaires, JB; Zacharias, W; Bumpous, JM; Garbett, NC [36]	2024	Thermal Liquid Biopsy Analysis of Saliva and Blood Plasma		Thermal Liquid Biopsy Analysis of Saliva and Blood Plasma	Saliva TLB profiles not only differ between healthy control and HNC groups but may also facilitate the identification of tumor location and its stage.
Rocchetti, F; Tenore, G; Macali, F; Vicidomini, T; Podda, GM; Fantozzi, PJ; Silvestri, V; Porzio, V; Valentini, V; Ottini, L; Richetta, AG; Valentini, V; Della Monaca, M; Grenga, C; Polimeni, A; Romeo, U [55]	2024	molecular based	Circulating microRNAs in Saliva and Plasma	saliva and plasma	Salivary miR-138/424 profiling distinguishes OPMD and OSCC; plasma levels are low, favoring saliva biopsy.
Ahmed, AA; Sborchia, M; Bye, H; Roman-Escorza, M; Amar, A; Henley-Smith, R; Odell, E; Mcgurk, M; Simpson, M; Ng, T; Sawyer, EJ; Mathew, CG [56]	2024	molecular based	mutations	saliva	NGS validated saliva-based detection of tumor mutations in 82% of OSCC cases.
Alafaria, HAA; Jalal, AS [57]	2024	molecular based	DNA methylation biomarkers	saliva	Salivary TRH, p16, MGMT, and RASSF1A methylation showed up to 100% sensitivity in tongue cancer.
Liu, K.Y.P., Ng, S., Taleghani, M., Zhu, S.Y., Carraro, A., Chen, Z., Palčič, B., Poh, C.F., Guillaud, M [6]	2024	molecular based	DNA ploidy approach	oral brush	Negative DOC results predicted no malignant progression for ≥3 years, supporting reliable risk stratification.
Hu Y, Xu M, Liu M, Peng H [18]	2025	molecular based	cell free RNA	blood and saliva	Salivary cfRNA profiling identified CLEC2B as an OSCC-specific biomarker, unlike plasma.
Lepper, TW; do Amaral, LN; Espinosa, ALF; Guedes, IC; Rönnau, MM; Daroit, NB; Haas, AN; Visioli, F; Neto, MMD; Rados, PV [26]	2025	artificial intelligence/molecular based	NOR	oral exfoliative cytological smears	AI-based AgNOR quantification (>3.69/nucleus) achieved 90% accuracy, improving standardization in OSCC screening.
Khayamzadeh, M; Ghaderian, SMH; Garajei, A; Jolani, MS; Tavassoli, A; Khodaee, P [58]	2025	molecular based	e ITGB8 and MIAT-lncRNA Expression	Salivary, Plasma, and Tissue	ITGB8 is a strong liquid biopsy biomarker for OSCC; MIAT shows moderate, tissue-blood–restricted diagnostic value.
Lian W, Lindblad J, Runow Stark C, Hirsch JM, Sladoje N [32]	2025	artificial intelligence		fluorescence whole slide microscopy imaging to analyze Papanicolaou stained liquid-based cytology slides of brush biopsies	Combining autofluorescence (FL) with BF microscopy enhances AI performance in early oral cancer detection.
Singh, S; Goyal, R; Gupta, A; Singh, R; Singh, M; Mehra, P; Pramanik, R; Suri, V; Ali, S [59]	2025	molecular based	Cell-free DNA	Liquid biopsy	In HNSCC, plasma cfDNA correlates with tumor stage, increasing progressively and peaking in Stage IV.
Hemavathy, OR; Ramaswamy, MM; Priya, CDM; Bhardwaj, S [60]	2025	molecular based	CD24 and CD44	Liquid Biopsy	CD24 is a dependable early diagnostic biomarker for oral cancer in tissue and blood.
Mohammad, S; Ullah, I; Jan, ZN; Khan, M; Aleem, B; Khattak, FA; Khan, AU; Bukhari, A; Khurram, A; Ali, A [61]	2025	molecular based	circulating tumor DNA	plasma	ctDNA dynamically predicts treatment response and prognosis in OSCC; higher levels indicate metastasis and poorer survival.
Kampel L, Tsuriel S, Trejo LL, Hadi Y, Horowitz G, Warshavsky A, Hershkovitz D, Muhanna N [62]	2026	molecular based	TP53-Mutated Tumor DNA	saliva and plasma	Tumor mutation detection in plasma and saliva supports surgical margin monitoring and complementary disease assessment.

## Data Availability

No new data were created or analyzed in this study. Data sharing is not applicable to this article.

## Supplementary Materials

The following supporting information can be downloaded at: https://www.mdpi.com/article/10.3390/diagnostics16091364/s1. The PRISMA-ScR checklist used for reporting this scoping review is provided as Supplementary File S1: PRISMA-ScR checklist. Reference [41] is cited in the Supplementary Materials.

## Abbreviations

The following abbreviations are used in this manuscript:

AgNOR	Argyrophilic Nucleolar Organizer Region
AI	Artificial Intelligence
cfDNA	Cell-Free DNA
cf-mtDNA	Cell-Free Mitochondrial DNA
ctDNA	Circulating Tumor DNA
DAPK1	Death-Associated Protein Kinase 1
DNA-ICM	DNA Image Cytometry
DSC	Differential Scanning Calorimetry
LBC	Liquid-Based Cytology
MIP	Molecularly Imprinted Polymer
miRNA	MicroRNA
NGS	Next-Generation Sequencing
NOR	Nucleolar Organizer Region
NUBP2	Nucleotide-Binding Protein 2
OPMD	Oral Potentially Malignant Disorder
OSCC	Oral Squamous Cell Carcinoma
PCC	Population–Concept–Context
PRISMA-ScR	Preferred Reporting Items for Systematic Reviews and Meta-Analyses Extension for Scoping Reviews
PYCR1	Pyrroline-5-Carboxylate Reductase 1
SERS	Surface-Enhanced Raman Spectroscopy
TK1	Thymidine Kinase 1
TGF-β1	Transforming Growth Factor Beta 1
ZNF471	Zinc Finger Protein 471
